# Efficiency of an Optimized Care Organization in Fibromyalgia Patients: The From Intent to Move (FIMOUV) Study Protocol of a Randomized Controlled Trial

**DOI:** 10.3389/fpubh.2021.554291

**Published:** 2021-05-25

**Authors:** Claire Colas, Julie Goutte, Christelle Creac'h, Luc Fontana, Marie-Pierre Vericel, Jessica Manzanares, Marie Peuriere, Madjid Akrour, Charly Martin, Emilie Presles, Nathalie Barth, Jessica Guyot, Maël Garros, Béatrice Trombert, Catherine Massoubre, Frédéric Roche, Léonard Féasson, Hubert Marotte, Pascal Cathebras, David Hupin

**Affiliations:** ^1^University Lyon, UJM-Saint-Etienne Sainbiose Laboratory, INSERM U1059, Saint-Étienne, France; ^2^Department of Clinical and Exercise Physiology, University Hospital Center, Saint-Étienne, France; ^3^Department of Internal Medicine, University Hospital Center, Saint-Étienne, France; ^4^Pain Center, University Hospital Center, Saint-Étienne, France; ^5^University Claude Bernard, Central Integration of Pain (NeuroPain) Lab—Lyon Neuroscience Research Center, INSERM U1028, CNRS, Bron, France; ^6^Department of Occupational and Environmental Medicine, University Hospital Center, Saint-Étienne, France; ^7^University Lyon, University Lyon 1, University St Etienne, University Gustave Eiffel, IFSTTAR, UMRESTTE, UMR_T9405, Saint-Étienne, France; ^8^Clinical Research, Innovation and Pharmacology Unit, University Hospital Center, Saint-Étienne, France; ^9^Department of Clinical Investigation Center, CIC 1408-INSERM, University Hospital Center, Saint-Étienne, France; ^10^University Lyon, UJM-Saint-Etienne Chaire Santé des Ainés, Saint-Étienne, France; ^11^Gerontopole Auvergne-Rhone-Alpes, Saint-Étienne, France; ^12^Sport Health House, CDOS 42, Saint-Étienne, France; ^13^Department of Public Health, University Hospital Center, Saint-Étienne, France; ^14^Department of Psychiatry, University Hospital Center, Saint-Étienne, France; ^15^University Lyon, UJM-Saint-Etienne Interuniversity Laboratory of Human Movement Biology, EA 7424, Saint-Étienne, France; ^16^Department of Rheumatology, University Hospital Center, Saint-Étienne, France; ^17^Department of Medicine, K2, Solna Karolinska Institutet, Stockholm, Sweden

**Keywords:** fibromyalgia, exercise, patient education, behavior in physical activity, accelerometry, care organization

## Abstract

**Introduction:** Fibromyalgia (FM) is characterized by multiple symptoms including pain, fatigue, and sleep disorders, altering patient's quality of life. In the absence of effective pharmacological therapy, the last European guidelines recommend a multidisciplinary management based on exercise and education. Thus, our main objective was to measure the effectiveness of a healthcare organization offering a specific program of adapted physical activity combined with a therapeutic education program for FM patients.

**Methods and Analysis:** The From Intent To Move (FIMOUV) study will recruit 330 FM patients randomized into two groups: test and control. The test group will benefit from a 1-month mixed exercise training program supervised at the hospital, followed by 2 months in a community-based relay in a health-sport structure. In addition, each of the two groups will benefit from therapeutic patient education sessions. The main endpoint is the measurement of the level of physical activity by accelerometry at 1 year. The secondary endpoints concern adherence to the practice of physical activity, impact on lifestyle, state of health, and physical capacity, as well as an estimate of the budgetary impact of this management strategy.

**Discussion:** This interventional research will allow us to assess the evolution of behaviors in physical activity after an FM syndrome management based solely on patient education or based on a supervised and adapted practice of physical activity associated with this same therapeutic education program. It seems to be the first study evaluating the impact of its intervention on objective data for measuring physical activity and sedentary behavior *via* accelerometry among FM patients.

**Trial registration:** ClinicalTrials.gov NCT04107948.

## Introduction

Fibromyalgia (FM) affects 2–5% of the adult population, with a feminine predominance (1). This syndrome is characterized by multiple symptoms including pain, fatigue, and sleep disorders, altering patient's quality of life (2). Physiopathology is still poorly understood, but a dysfunction of the central nervous system is evoked, involving various mechanisms of pain pathways (3). Diagnosis of FM is based on the American College of Rheumatology criteria (4, 5) and associates a quantitative pain rating scale (Widespread Pain Index) and a rating scale of the severity of symptoms associated with pain (Symptom Severity Scale). According to these last recommendations (4), FM diagnosis is no more an elimination diagnosis but rests on the scores of these two scales, the chronicity of pain (>3 months), the location of pain (>4/5 regions) and does not exclude an association with another pathology (with lesion or organ damage).

Actually, no treatment can cure FM. Pharmacological management often remains on the prescription of analgesics, antidepressants, and/or antiepileptics, but these therapies are not always effective and often cause undesirable effects (6, 7). Nonpharmacological therapies should be advocated as a first-line treatment for patients suffering from FM, such as the practice of a regular physical activity, exercise being “the only ‘strong for' therapy-based recommendation in the [European League Against Rheumatism, EULAR] guidelines” (8).

Exercise has clinical relevance over several parameters such as fatigue, physical function, and quality of life (9). However, it can be applied by several ways of training. The American College of Sport Medicine (ACSM) elaborates the recommendations in terms of physical activity, applying the frequency, intensity, time, type, volume, and progression principle (10). For FM, there are no precise recommendations.

Endurance training is recommended by different medical societies for FM management (8, 11–14) in the range of two to three sessions a week, allowing to respect a rest period and avoid exacerbation of symptoms, at least for a period of 4–6 weeks in order to see a decrease in symptoms (15, 16) and a change in autonomic function (17). A light-intensity aerobic practice (15, 18) does not allow a decrease in symptom intensity, rather indicating a moderate to vigorous intensity physical activity (MVPA) practice. While high-intensity interval training (HIIT) is increasingly used in several pathologies (19–21), it is only minimally studied in FM. Demonstrating improvements in physical capacities (22, 23), HIIT would also be effective for decrease in fatigue (24, 25), pain tolerance (26), and opioid system (27, 28)—three major issues in FM syndrome—without change in pain or disease activity in patients with rheumatic disease (21). Strength training is less documented than aerobic training. Some studies have worked on weight resistance training (two weekly sessions during 16 weeks) with a progressive load from 50 to 80% of the one-repetition maximum, demonstrating a good compliance and an involvement of force correlated to a decrease of 39% in pain perception (29). This decrease in pain was also measured in a resistance program followed for 8 weeks with three sessions a week (30). Recently, Andrade et al. (17) identified a correlation between resistance training and reduction in psychological symptoms such as anxiety and depression. Muscle stretching and relaxation exercises also showed an impact on pain (31), pain perception and tolerance (32), quality of life, depression, and anxiety (33) in FM patients.

At equal time of activity, Sanudo et al. (34) noticed additional health benefits of a combined training program compared to a single modality program. A mixed training program is defined as combining at least two types of exercises among aerobics, resistance and flexibility training, and demonstrated an interest in quality of life (+7%), fatigue (−13%), and physical function (+11%), translating the clinical relevance of mixed exercise training for adults with FM (9).

Despite the absence of accurate recommendations on training modalities, as defined by the ACSM, a personalized and mixed training seems to be the best practice according to the EULAR recommendations (8), and it is also this type of training that is recommended for people suffering from chronic pain (35). In France, there are no professional recommendations of good practice for the management of FM. The health insurance recommends to physicians to give advice for the progressive upturn of physical activity for each person with FM, “according to its means.”

However, European and French recommendations agree to promote patient education to increase “understanding of the complex nature of the interactions between neurobiological processes, behaviors, and symptoms (36).” It showed beneficial impact on pain and pain catastrophizing (37) despite the different educational strategies. Moreover, an education program would be all the more effective if it is combined with an adapted physical activity (APA) program (38, 39).

Then, the problem is to know if simple advice for physical activity is sufficient (as recommended by the French health insurance and commonly performed in consultation) or if an adapted and supervised physical activity (as recommend by the EULAR) would not be more beneficial as a first intention. Thus, in this study, we want to optimize the care pathways of patients suffering from FM by offering a supervised APA program added to therapeutic patient education sessions. This is a prospective, multicenter, controlled, randomized, open-label intervention in two groups. The primary objective of this study is to measure the effectiveness of a healthcare organization offering APA for FM patients through an objective measure of physical activity at 1 year (average measured from 7 days of accelerometry). The protocol has been written according to the Standard Protocol Items: Recommendations for Interventional Trials guidelines.

## Methods and Analysis

### Study Setting

**Figure 1:**
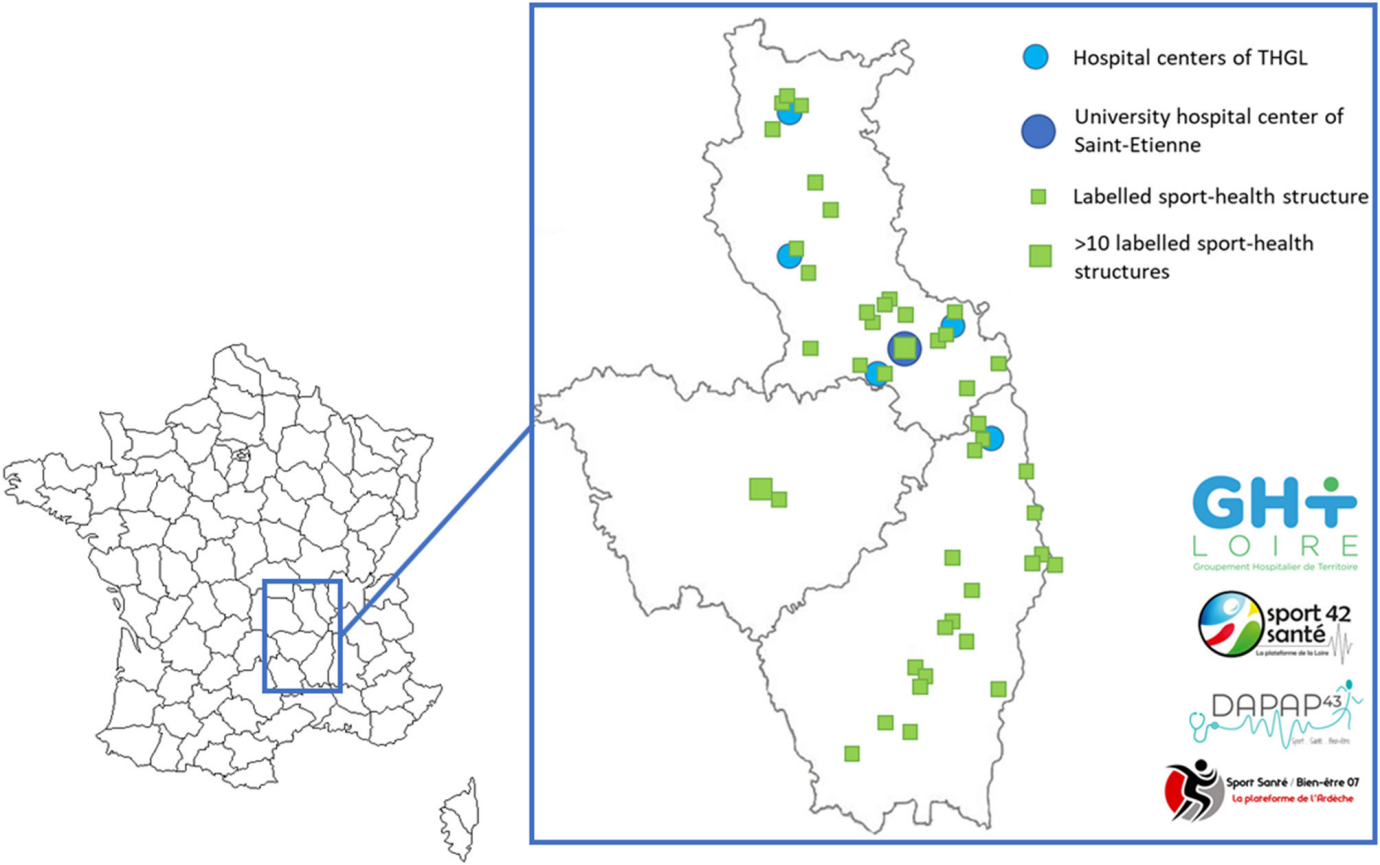
Project collaborators mapping. THGL: Territorial Hospital Group of Loire.

**Figure 2:**
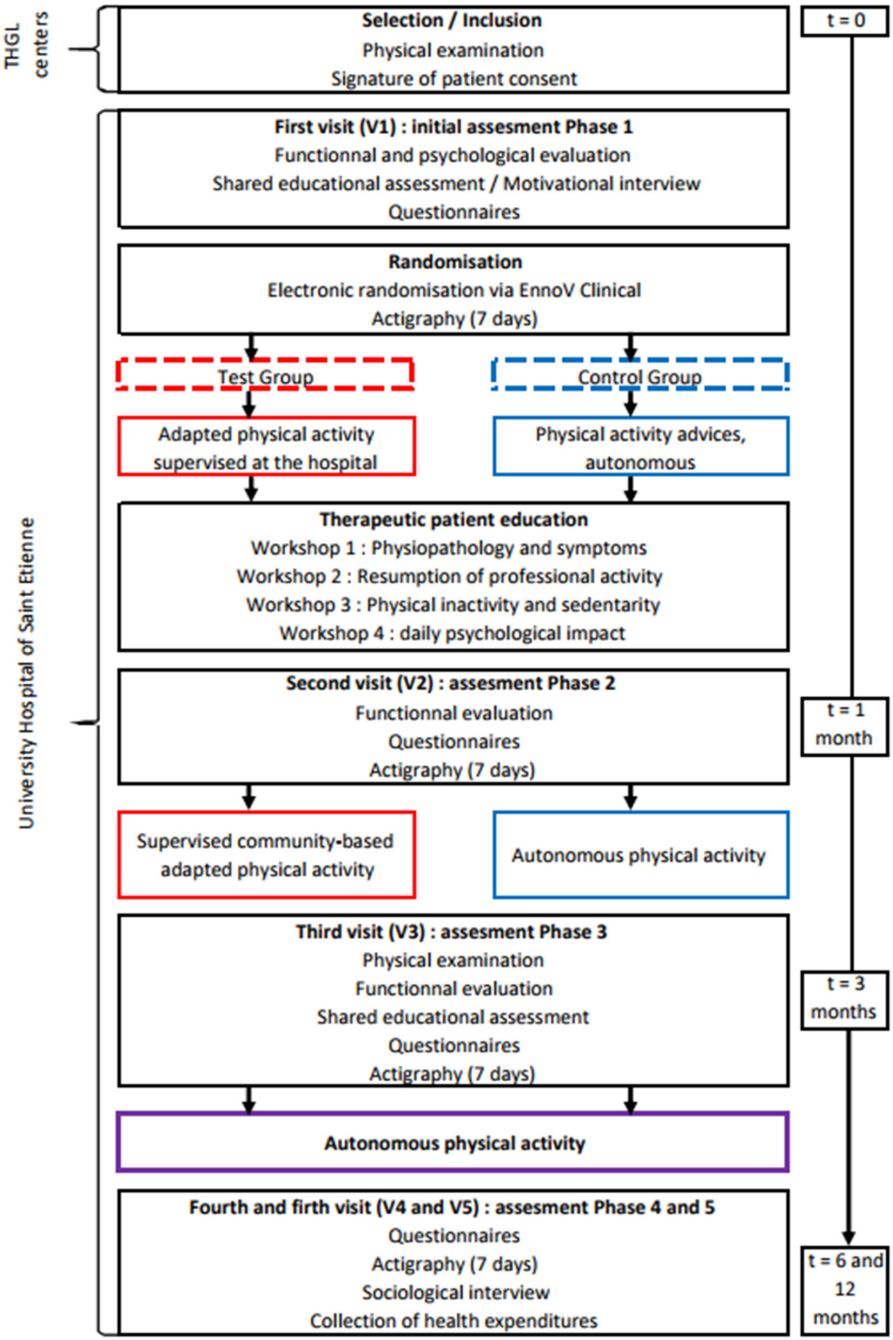
Study design of FIMOUV. THGL, Territorial Hospital Group of Loire.

The FIMOUV study is a collaboration between health professionals of the Territorial Hospital Group of Loire centers in France and physical activity professionals on this territory (Figure 1). Patients will be selected at their follow-up consultation, during which the doctor will give the information leaflet and the consent form. FM patients will then be summoned at the University Hospital of Saint-Etienne (Loire) for the inclusion visit (V1). Inclusion and exclusion criteria are detailed in Table 1. We want to compare a supervised training program to physical activity advice, both benefiting from four therapeutic education sessions. Patients are then followed for 1 year (Figure 2).

**Table 1:** Eligibility criteria.

Inclusion criteria	Patient over 18 years old
	FM syndrome according to ACR criteria (total score WPI + SSS ≥ 12)
	Language skills in oral and written French
	Sedentary or low level of activity (<150 min of regular physical activity per week at the time of inclusion)
	Affiliation to a social security scheme
	Signing informed consent
Exclusion criteria	Cardiac or respiratory diseases that contraindicate the practice of physical activity
	Severe comorbidities contraindicating the practice of physical activity (rhythm disorders, severe obstructive respiratory failure, gonarthrosis, etc.)
	Impossibility of submitting to medical monitoring of the program for geographical, social, or psychological reasons
	Patient deprived of liberty or patient under guardianship

*FM, fibromyalgia; ACR, American College of Rheumatology; WPI, Widespread Pain Index; SSS, symptom severity scale*.

### Interventions

#### Training Program

##### Test Group

**Figure 3:**
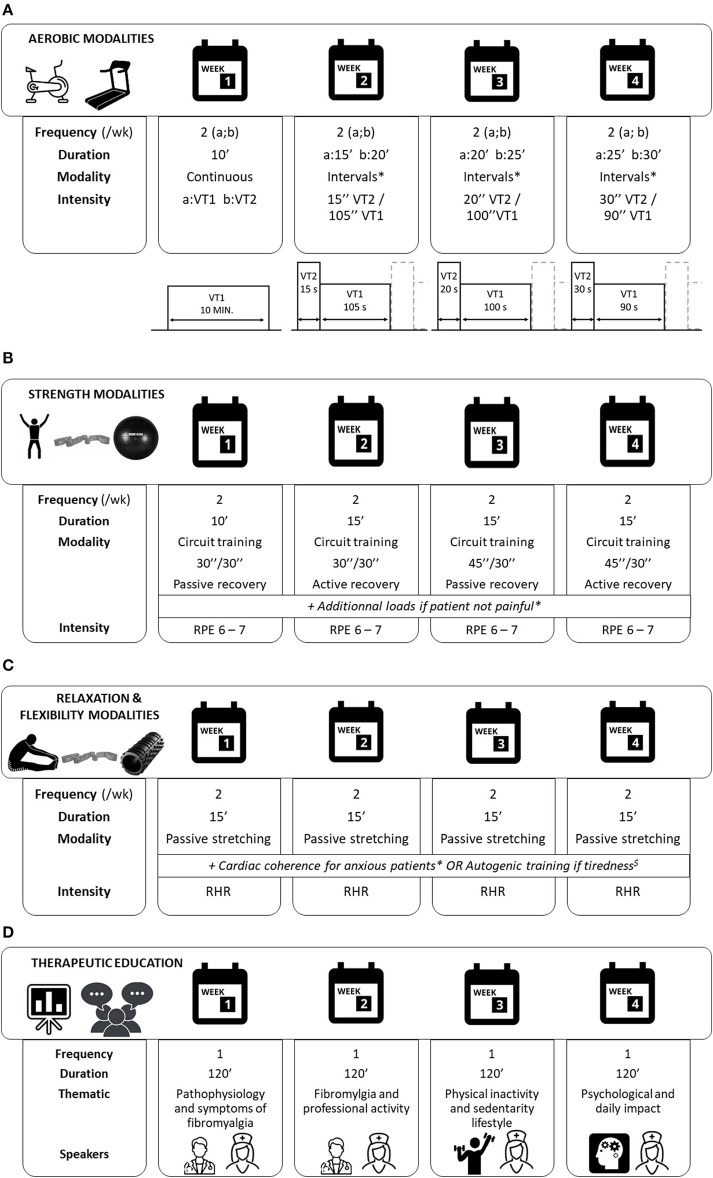
FIMOUV program intervention. **(A)** Modalities of aerobic training during the first month of intervention. Each session starts with 10 min warmup and ends with 10 min cooldown. *A continuous training at VT1 can be proposed if the subject's physical capacity does not allow the increase in workloads, as they are made on interval training. (a;b) corresponds to each session of the week; VT1: ventilatory threshold 1; VT2: ventilatory threshold 2. **(B)** Modalities of strength training during the first month of intervention. *Load maximum 50% of 1-repetition maximum if pain evaluation <4/10 at the start of the session. RPE: rating of perceived exertion, Borg modified scale. **(C)** Modalities of relaxation and flexibility training during the first month of intervention. *Hospital Anxiety and Depression Scale >8 for anxiety item. ^$^Fatigue evaluation >7/10 at the start of the session. RHR: resting heart rate. (D) Modalities of therapeutic education program during the first month of intervention.

During the first month, the test group benefiting from two weekly exercise sessions of 90 min was supervised by an APA teacher during four consecutive weeks at the hospital. We propose a mixed training program (details on Figure 3), normalized and personalized to each patient profile, as follows:

– Endurance training (Figure 3A): progressive work toward an interval training at an intensity determined during a preliminary cardiorespiratory test (first and second ventilatory threshold, VT1/VT2) and monitored with a heart rate belt connected by Bluetooth (Polar H7, Polar®, Kempele, Finland). A continuous training at VT1 can be proposed if the subject's physical capacity does not allow an increase in workloads as they are made on interval training.
– Strength training: whole body circuit training with body weight or small equipment, with at least one exercise soliciting balance, coordination, and/or velocity. Additional loads representing a maximum 50% of one repetition maximum can be added in case of pain evaluation lower than 4/10 on the Borg modified visual analog scale (VAS) at the start of the session. Modalities of training are detailed in Figure 3B.
– Stretching and relaxation training (Figure 3C): according to the patient's profile, alternation between passive stretching, cardiac coherence for patients with a high levels of anxiety (measured during V1 thanks to the Hospital Anxiety and Depression scale, HADS), and autogenic training initiation if the fatigue evaluation is higher than 7/10 at the start of the session.

From the second to third months, each patient is addressed to a relay structure affiliated to the “Sport-Health Houses” of their department to continue a training program for one to two sessions of 60–90 min weekly. Certified by the Ministry of Sports, these health-sport platforms aim to facilitate the maintenance of physical activities among people experiencing health difficulties, with an individualized follow-up. Additionally, a training follow-up booklet will be delivered to ensure the continuity and attendance of training sessions (supervised and autonomous) and evaluate their efficacy on pain and fatigue symptoms thanks to the VAS.

##### Control Group

The control group receives advice and recommendations for independent physical activity practice at home in order to reach 30 min of moderate intensity physical activity, 5 days a week, as recommended by the World Health Organization (WHO) (current clinical practice).

#### Therapeutic Education Program

Therapeutic patient education programs aim to acquire self-care skills and mobilize or acquire psychosocial skills. In this protocol, we propose four sessions of 2 h, in groups of three to eight patients. The two groups benefit from (1) one session about physiopathology and symptoms of FM by the internal medicine service, (2) one session on physical inactivity and sedentary lifestyle by the myology unit team, (3) one session on the resumption of professional activity by the occupational health service, and (4) one session about the psychological impact and daily routine by the pain center (Figure 3D). All sessions are driven by the coordinating nurse who makes the link between patients and the other health professionals. An individual therapeutic education session will be proposed at V1 and V3 *via* motivational interviewing led by one of the investigator doctors and the nurse/APA teacher duo in order to optimize the durability of the practice of regular physical activity.

### Assessment Measures

The primary outcome of interest will be the long-term therapeutic adherence of FM patients evaluated from an objective measure of physical activity (mean measured from 7 days of accelerometry; Actigraph GT3x, Pensacola, Florida, USA) 1 year after the setup of the organization of care offering initiation and support of APA during 3 months. This sustainability will result in a level of physical activity ≥7.5 MET-h/week at 1 year, corresponding to 150 min of moderate intensity physical activity per week, which is consistent with WHO recommendations.

The secondary objectives will evaluate the impact on the lifestyle (average time of physical activity and sedentary behavior, measured by accelerometry during 7 days), health status, fatigue, sleep, psychic distress, muscular strength and endurance, physical capacity, tolerance to pain and fatigue during training sessions, membership to physical activity, evaluation of the differential cost-effectiveness ratio, and finally an estimate of the budgetary impact of this care strategy.

Details and timetable are shown in Table 2.

**Table 2:** Assessment measures timetable.

	**Inclusion visit V1**	**Intervention 1st month**	**Post-intervention visit V2**	**Intervention 2nd and 3rd months**	**Post-intervention visit V3**	**Intermediate visit V4**	**Final visit V5**
**Screening**							
Informed consent	✓						
Medical consultation	✓						
Anthropometry	✓		✓		✓	✓	✓
Pain VAS	✓		✓		✓	✓	✓
Fatigue VAS	✓		✓		✓	✓	✓
MINI	✓						
MCM/Randomization	✓						
**Questionnaires**							
PGIC	✓		✓		✓	✓	✓
APAQ	✓		✓		✓	✓	✓
FSS	✓		✓		✓	✓	✓
HADS	✓		✓		✓	✓	✓
PCS	✓		✓		✓	✓	✓
Pittsburgh	✓		✓		✓	✓	✓
**Accelerometry**	✓		✓		✓	✓	✓
**Cardiorespiratory capacities**							
ECG	✓		✓		✓		
RFE	✓		✓		✓		
VO_2_max	✓		✓		✓		
**Muscular capacities**							
Isometric strength^*^	✓		✓		✓		
Dynamic strength^$^	✓		✓		✓		
Endurance strength^$^	✓		✓		✓		
**EXPLORATION OF THE ANS**							
Baroreflex	✓		✓		✓		
**EXERCISE SESSIONS**							
Pain and fatigue tolerance (VAS)		✓		✓			
PA adhesion			✓		✓	✓	✓
**Therapeutic education**							
Educational diagnosis	✓				✓		
TPE sessions		✓					
**Health expenditure reporting**						✓	✓

*VAS, visual analog scale; MINI, mini international neuropsychiatric interview; MCM, multidisciplinary consulting meeting; PGIC, patient global impression of change; APAQ, adult physical activity questionnaire; FSS, fatigue severity scale; HADS, hospital anxiety and depression scale; PCS, pain catastrophizing scale; ECG, electrocardiogram; RFE, respiratory functional exploration; VO_2_max, maximum oxygen consumption; ANS, autonomic nervous system; PA, physical activity; TPE, therapeutic patient education*.

* *arms and legs*.

$ *legs*.

### Sample Size

Population size is calculated on an expected difference of 20% of the level of MVPA (≥3 METs for metabolic equivalent tasks). Estimating that at baseline, patients are at 3.9 MET-h/week with a standard deviation between 2 and 4 (40), for an alpha risk of 5% and a potency set at 90%, we need to include 150 patients per group; adding an expected dropout rate of 10%, it is necessary to include 165 patients per group to meet the main goal, which means a total of 330 patients.

### Randomization and Data Collection

Randomization and data collection will be centralized *via* the EnnoV Clinical platform (electronical randomization, Paris, France) and filled in by the referral doctor and the nurse/APA teacher duo. Only investigators and technicians of the study have access to these data.

Patients will be randomized after a multidisciplinary consulting meeting carried out in the presence of the investigators of the study, the referring doctor and, if necessary, with their general practitioner, before the program starts. This meeting aims to decide about the optimal care for the patient. It will be decided if the patient can follow an exercise training. In case of contraindication, the patient will not be randomized into the study.

### Statistical Analysis

The included population will first be described globally and in groups (test or control), the comparability of the groups will be verified. For quantitative variables, Student's *T*-tests or rank tests (in case of non-normal distribution) will be implemented. The normality of the variables will be checked beforehand with a Shapiro-Wilk test. For qualitative variables, chi-square or Fisher-exact tests (if theoretical numbers are insufficient) will be used. The results will be considered significant at the 5% threshold. The analysis will be carried out in intent-to-treat. An analysis of the main endpoint will be done per protocol. Patients who do not perform 85% of the exercises will be excluded from per-protocol statistical analysis. In addition, we will lead a qualitative study through semistructured individual interviews conducted by a sport and health sociologist. Thirty patients will be recruited in each of the two groups (41, 42). A full transcript will be followed by a thematic and comparative analysis of the cross-data content.

## Discussion

This study aims to evaluate the efficacy of an optimized care organization within the Loire Territory Hospital Group, illustrated by the sustainability of therapeutic adherence of FM patients. This interventional research will allow us to assess the evolution of behaviors in physical activity after a FM syndrome management based solely on patient education or based on a supervised and adapted practice of physical activity associated with this same therapeutic education program.

Supervised exercise allows a better participation and adhesion to physical activity in sedentary women (43) and an improvement in psychological well-being determined by the result of HADS in FM (44). Improvements up to two times greater than those of unsupervised training can be observed, however without necessarily being maintained for the long-term at 1 year (45). A recent study has explored the experience of the transition from a supervised exercise in the hospital to a community-based unsupervised program in prostate cancer patients, emphasizing the importance of a structured, accompanied and over time monitored relay (46). Dnes et al. (47) identified the barriers and facilitators to participating in community-based exercise opportunities from the perspective of adults with chronic pain, including FM. The main factors identified were participation in groups with similar levels of capabilities, delivered by an instructor able to tailor exercises to chronic pain and recommended by their healthcare provider. Also, in this project, the implementation of physical activity in community programs, chosen by the patient himself/herself according to his/her preferences and availability, could strengthen the integration of physical activity into daily life through the connection with the sports health centers, which have the role of labeling the health-sports club, with groups specific to chronic pathologies delivered by trained instructors.

The combination of education and physical activity demonstrated its efficacy on management of several FM symptoms. However, the level of physical activity practice in the short-term after the intervention and at the long-term at 1 year has been barely studied. Physical activity monitors are interesting tools to better understand the role of physical activity in rheumatic disease populations (48). For example, the use of a pedometer to measure the activity in FM patients after a multidisciplinary intervention based on cognitive behavioral therapy and physical therapy demonstrated an increase in exercise capacity and regularity (49). Others studies evaluated the physical activity behavior in FM patients compared to healthy subjects, and the link between their behavior and their symptoms (40, 50). However, to our knowledge, no study has evaluated the impact of its intervention on objective data for measuring physical activity and sedentary behavior *via* accelerometry among FM patients.

Qualitative analysis of the project will provide data for evaluation of the acceptability and the feasibility of our intervention, according to the patients' experiences. This analysis will allow us to better understand the barriers and facilitators that can be used to design an intervention more suited to the expressed needs of the participants.

To conclude, the multidisciplinary management proposed must contribute to the construction of a solid therapeutic alliance between the patient and the management of its FM syndrome and thus give the initial medical prescription of APA the appearance of a therapeutic project. It is also a question of strengthening the city–hospital link by (1) developing an optimal care sector, (2) which combines therapies validated by scientific societies, (3) including APA that we wish to place at the center of a multidisciplinary and consensual care organization, and (4) which relies on existing and efficient structures for many chronic pathologies, with (5) an objective of sustainability within the largest national territory hospital group.

## Ethics Statement

The protocol is in accordance with ethical principles established by the 18th World Medical Assembly (Helsinki 1964) and was approved by the institutional review board (CPP Sud-Méditerranée II, France). Each patient will be awarded an information notice explaining the study and must sign an informed consent form before being able to participate in the research.

## Author Contributions

The FIMOUV investigators from University Hospital of Saint-Etienne contributed to the conception and design of the study. CCo, M-PV, MA, and DH contributed to the acquisition of data. CCo wrote the first draft of the manuscript. DH provided critical revision for intellectual content and oversight. All the authors reviewed and approved the final version of the manuscript.

## Conflict of Interest

The authors declare that the research was conducted in the absence of any commercial or financial relationships that could be construed as a potential conflict of interest.
